# Tissue Destruction in Bullous Pemphigoid Can Be Complement Independent and May Be Mitigated by C5aR2

**DOI:** 10.3389/fimmu.2018.00488

**Published:** 2018-03-15

**Authors:** Christian M. Karsten, Tina Beckmann, Maike M. Holtsche, Jenny Tillmann, Sabrina Tofern, Franziska S. Schulze, Eva Nina Heppe, Ralf J. Ludwig, Detlef Zillikens, Inke R. König, Jörg Köhl, Enno Schmidt

**Affiliations:** ^1^Institute of Systemic Inflammation, University of Lübeck, Lübeck, Germany; ^2^Lübeck Institute of Experimental Dermatology (LIED), University of Lübeck, Lübeck, Germany; ^3^Department of Dermatology, University of Lübeck, Lübeck, Germany; ^4^Institute of Medical Biometry and Statistics, University of Lübeck, Lübeck, Germany; ^5^Division of Immunobiology, Cincinnati Children’s Hospital and College of Medicine, University of Cincinnati, Cincinnati, OH, United States

**Keywords:** autoantibody, dermal–epidermal junction, BP180, type XVII collagen, skin, treatment

## Abstract

Bullous pemphigoid (BP), the most frequent autoimmune bullous disorder, is a paradigmatic autoantibody-mediated disease associated with autoantibodies against BP180 (type XVII collagen, Col17). Several animal models have been developed that reflect important clinical and immunological features of human BP. Complement activation has been described as a prerequisite for blister formation, however, the recent finding that skin lesions can be induced by anti-Col17 F(ab′)_2_ fragments indicates complement-independent mechanisms to contribute to blister formation in BP. Here, *C5*^−/−^ mice injected with anti-Col17 IgG showed a reduction of skin lesions by about 50% associated with significantly less skin-infiltrating neutrophils compared to wild-type mice. Reduction of skin lesions and neutrophil infiltration was seen independently of the employed anti-Col17 IgG dose. Further, *C5ar1*^−/−^ mice were protected from disease development, whereas the extent of skin lesions was increased in *C5ar2*^−/−^ animals. Pharmacological inhibition of C5a receptor 1 (C5aR1) by PMX53 led to reduced disease activity when applied in a prophylactic setting. In contrast, PMX-53 treatment had no effect when first skin lesions had already developed. While C5aR1 was critically involved in neutrophil migration *in vitro*, its role for Col17-anti-Col17 IgG immune complex-mediated release of reactive oxygen species from neutrophils was less pronounced. Our data demonstrate that complement-dependent and -independent mechanisms coexist in anti-Col17-autoantibody-mediated tissue destruction. C5aR1 and C5aR2 seem to play opposing roles in this process with C5aR1 exerting its primary effect in recruiting inflammatory cells to the skin during the early phase of the disease. Further studies are required to fully understand the role of C5aR2 in autoantibody-mediated skin inflammation.

## Introduction

Type XVII collagen (Col17), also termed BP180, is a component of the dermal–epidermal junction (DEJ) and target antigen in various subepidermal blistering autoimmune disorders, the most frequent being bullous pemphigoid (BP) (1–4). The majority of BP sera reacts with epitopes clustered within the 16th non-collagenous (NC16A) domain of Col17 (5). Due to the relatively low homology between human NC16A and the corresponding murine NC15A domain, direct evidence for the functional relevance of antihuman (h)Col17 was only successful after Nishie et al. had expressed *hCOL17* in mice (6, 7). Before, the passive transfer of rabbit antibodies generated against murine and hamster Col17 in neonatal animals had resulted in a subepidermal blistering phenotype mimicking important immunopathological signs of human BP (8, 9).

The neonatal mouse model based on the passive transfer of rabbit antimurine collagen type XVII (anti-mCol17) IgG has vigorously been explored by the group of Liu and Diaz that highlighted the pathogenic importance of, e.g., complement activation, inflammatory cells such as neutrophils, mast cells, and macrophages, and the release of proteolytic enzymes at the DEJ in this model [(8), reviewed in Ref. (10)]. Of note, complement activation appeared to be pivotal in this model as shown by several lines of evidence: *C5*- and *C4*-deficient (^−/−^) mice were completely protected against the pathogenic effect of anti-mCol17 IgG, and pharmacological inhibition of C1q as well as complement depletion by cobra venom prevented skin lesions in the neonatal mouse model of BP (11, 12). Furthermore, in this model, factor B (*CFB*)^−/−^ mice developed delayed and less intense blistering and the C5a receptor 1 (C5aR1) on mast cells was shown to be critical for the formation of skin lesions (12, 13). In addition, mutated non-C1q-binding anti-hCol17 IgG1 was not capable to induce BP lesions in neonatal *COL17*-humanized mice (14). More recently, however, in neonatal mice, the injection of F(ab′)_2_ fragments of BP patients’ IgG and rabbit anti-hCOL17 IgG resulted in skin fragility questioning the impact of complement activation for lesion formation in BP (15).

To further clarify the role of complement activation in the pathogenesis of BP and to explore the potential therapeutic use of complement inhibitors that are increasingly being developed (16), we made use of a recently established passive transfer model of BP in adult mice (17). In this model, the injection of rabbit IgG generated against the murine homolog (amino acids 497–573) of the immunodominant human NC16A domain of BP180 in adult C57BL/6 or Balb/c mice on days 2, 4, 6, 8, and 10 triggered an inflammatory reaction that mimicked major characteristics of the human disease including (i) complement-fixing IgG along the DEJ, (ii) spontaneous erythema and erosions arising from day 4, (iii) subepidermal blisters by histopathology, and (iv) lesional infiltration of neutrophils and eosinophils. Furthermore, skin lesions develop over some days and thus, this model is suitable to study anti-inflammatory mediators in a quasitherapeutic setting, i.e., in mice with already established skin lesions (17).

Here, the extent of BP skin lesions was markedly reduced in *C5*^−/−^ mice as compared to wild-type mice and pathogenicity was mediated by C5aR1, while C5aR2 was protective. Pharmacological inhibition of C5aR1 as well as *in vitro* analyses indicated that complement activation may exert its major pathophysiological impact in the early phase of the disease through the regulation of neutrophil accumulation in the skin.

## Materials and Methods

### Mice

C57BL/6J, Balb/c, *Fcer*^−/−^ mice (B6;129P2-*Fcer^tm1Rav^*/J), *C5ar1*^−/−^ (on C57BL/6 background), and *C5ar2*^−/−^ mice (for *in vivo* experiments on Balb/c background, for *in vitro* experiments on C57BL/6 background) were bred and housed at 12 h light–dark cycle at the experimental animal facility in the University of Lübeck. *C5*^−/−^ mice (B10.D2-*Hc^0^H2^d^H2*-*T18^c^*/oSnJ) and the corresponding wild-type controls (B10.D2-*Hc^1^H2^d^H2-T18^c^*/nSnJ) were obtained from Jackson Laboratories (Bar Harbor, ME, USA). All injections and bleedings were performed on eight to twelve-week old mice narcotized by intraperitoneal (i.p.) injection of a mixture of ketamine (100 µg/g) and xylazine (15 µg/g).

Animal experiments were approved by the Animal Care and Use Committee of Schleswig-Holstein (Kiel, Germany; 21-2/11, 40-3/15) and performed by certified personnel.

### Generation and Characterization of Rabbit Antibodies to mCol17

The extracellular portion of the 15th non-collagenous domain (NC15A) of mCol17 covering the stretch directly adjacent to the transmembrane domain (amino acids 497–573) was expressed as glutathione-*S-*transferase (GST) fusion protein and purified by affinity chromatography as previously described (18). Pathogenic anti-mCol17 IgG was generated in New Zealand white rabbits as reported (17, 19). Normal rabbit serum was obtained from CCPro (Oberdorla, Germany).

### EndoS Preparation and IgG Hydrolysis *In Vitro*

Pretreatment of rabbit IgG was performed as previously described (20, 21). One milligram of rabbit anti-mCOL17 IgG was incubated with 5 µg recombinant GST-EndoS in PBS for 16 h at 37°C followed by affinity removal of GST-EndoS by serial passages over Glutathione-Sepharose 4B columns (GE Healthcare, Uppsala, Sweden). IgG hydrolysis was verified by SDS-PAGE and lectin blot analyses as previously described (21).

### Passive Transfer Mouse Model

Affinity-purified rabbit anti-mCol17 IgG and normal rabbit IgG, respectively, was injected subcutaneously into the neck of mice every second day over a period of 12 days at individual doses of 7.5 or 10 mg/ml IgG unless stated otherwise. At the time of IgG injections, mice were weighed and examined for their general condition and evidence of cutaneous lesions (i.e., erythema, blisters, erosions, and crusts). Cutaneous lesions were scored as involvement of the skin surface as previously described (17, 22). At day 12, all mice were sacrificed, blood was taken, and both lesional and perilesional biopsies were taken for histopathological analysis (stored in 4% buffered formalin) and direct IFM (stored at −80°C), respectively. C5a receptor antagonist PMX53 was provided by Dr. Trent Woodruff (University of Queensland, Brisbane, Australia). 20 µg of PMX53 per mouse were daily injected i.p. from days 0 to 11 and from days 4 to 11, respectively.

### Immunofluorescence Microscopy and Histopathology

Tissue-bound autoantibodies and complement deposits were detected by direct IF microscopy of frozen sections using FITC-conjugated rabbit antimouse IgG (1:100; Dako, Hamburg, Germany), FITC-conjugated murine antimouse C3 (1:50; Cappel, MP Biomedicals, Solon, OH, USA), and murine antimouse C5 antibody (1:100; Cell Sciences, Canton, MA, USA) detected by FITC-labeled rabbit antimouse IgG (1:100; Dako). Staining was evaluated using a Keyence BZ-9000 microscope (Keyence, Neu-Isenburg, Germany) and quantified using ImageJ (http://rsbweb.nih.gov/ij/) software. Formalin-fixed skin samples were processed into paraffin blocks. Four micrometer sections were stained with hematoxylin and eosin according to standard protocols.

### Neutrophil-Specific Myeloperoxidase (MPO) Activity

Myeloperoxidase activity, corresponding to the total granulocyte infiltration, was assessed in homogenized ear-specimens as described in previous protocols (23). MPO content was expressed as units of MPO activity per milligram of protein. Protein concentrations were determined by a dye binding assay (Thermo Scientific, Rockford, IL, USA) using bovine serum albumin as a standard.

### Neutrophil Preparation

Mouse neutrophils were purified as previously described (24). Briefly, bone marrow cells from femurs and tibiae were flushed, red blood cells lysed with hypotonic NaCl and cells were separated by 62.5% Percoll^®^ (GE Healthcare, Uppsala, Sweden) gradient. For higher purity of neutrophils, T- and B-cells were depleted by MACS separation with anti-CD3ε and anti-CD19 antibodies (Miltenyi Biotech, Bergisch Gladbach, Germany). Purity of neutrophils was consistently >90% as determined by FACS analysis.

### Reactive Oxygen Species (ROS) Release Assay

In this assay, intra- and extracellular ROS of neutrophils was measured using luminol-amplified chemiluminescence after incubation with immune complexes and various controls, respectively, as described previously (25, 26). In brief, 96-well plates were coated with 20 mg/ml of mCol17 NC15A overnight. After blocking with PBS containing 1% BSA and 0.05% Tween-20 (PBS-T), 1 mg/ml rabbit anti-mCol17 IgG was incubated for 1 h at 37°C. After washing with PBS-T untreated or heat-inactivated (30 min, 56°C) mouse serum or recombinant C5a (R&D Systems Inc., Minneapolis, MN, USA) diluted in 100 µl of chemiluminescence medium were added to the immune complexes. 100 µl of cell suspension (2 × 10^6^/ml) containing 60 µg/ml of luminol (5-amino-2,3-dihydro-1,4-phthalazindione; Sigma Aldrich, Hamburg, Germany) was given to each well and generation of ROS was determined in a microplate luminometer (Wallac 1420 VICTOR TM, Perkin Elmer, Waltham, MA, USA). Chemiluminescence data were expressed as relative light units. Data are derived from at least two independent experiments performed in quadruplicates.

### Migration Assay

Chemotaxis of bone-marrow-derived cells was performed as described previously (27). Briefly, bone-marrow neutrophils were collected as described above and then resuspended in chemotaxis medium (HBSS containing 2% BSA) at a density of 5 × 10^6^ cells/ml. As a chemoattractant, we used C5a (12.5 nM; Hycult, Uden, Netherlands), which was diluted in chemotaxis medium. The chemoattractant was placed in the bottom wells of a modified Boyden chamber (Neuro Probe, Gaithersburg, MD, USA) and overlaid with a 3 µm polycarbonate membrane. Then, 50 µl of the cell suspension were placed in the top wells of the assembled Boyden chamber and incubated at 37°C in 5% CO_2_ for 30 min. Subsequently, the membranes were removed and the cells on the bottom of the membrane stained with Diff-Quick (Merck, Darmstadt, Germany). The numbers of migrated cells for each well were counted in five different high-power fields and the number of cells per mm^2^ was calculated by computer-assisted light microscopy. Results are expressed as the mean value of triplicate samples of at least two independent set of experiments.

### Statistical Analysis

Sigma plot 11.0 (Systat Software Inc., Chicago, IL, USA) and R version 3.4.0 (April 21, 2017) (http://www.r-project.org/) were used to perform statistical analyses. *p*-Values were determined by Mann–Whitney *U*-tests for comparisons between two independent groups, Kruskal–Wallis tests for comparisons between more than two independent groups, nd non-parametric analyses of variance for comparisons including different time points. For the latter, we employed the R package nparLD (28).

## Results

### Complement Deposition at the DEJ Does Not Correlate with Disease Activity in Experimental BP

**Figure 1:**
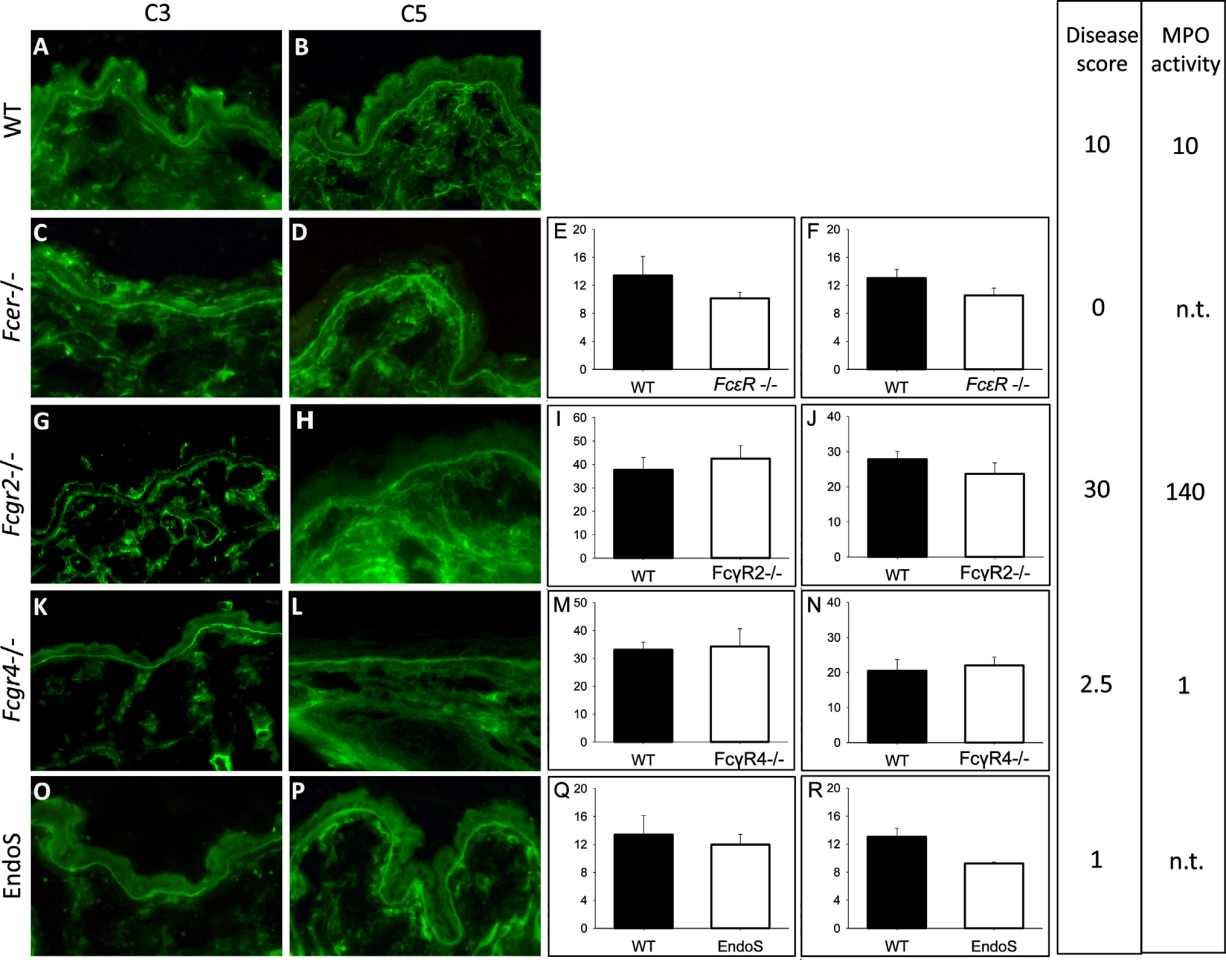
Complement activation at the dermal–epidermal junction (DEJ) is not related to the extent of clinical disease in experimental bullous pemphigoid. The intensity of C3 **(E,I,M,Q)** and C5 deposition **(F,J,N,R)** at the DEJ did not differ between wild-type (WT; **A,B**) mice, mice deficient of the FcRγ [*Fcer*^−/−^; **(C,D)**], FcγRIIB [*Fcgr2b*^−/−^; **(G,H)**], FcγRIV [*Fcgr4*^−/−^; **(K,L)**], and EndoS-treated animals **(O,P)** as quantified using Image J software. Furthermore, the amount of complement deposition was not reflected by the extent of skin lesions/disease score and the myeloperoxidase (MPO) activity determined in ear skin. Data for disease activity were assembled from different experiments with five mice in each group done at different time points (17). A and B are representative pictures selected from one of the experiments. For direct comparison, disease activity and MPO activity are indicated in arbitrary units related to the mean clinical score and MPO activity in WT animals in each experiment set to 10. n.t., not tested.

Adult mice lacking the activating γ-chain of the Fc receptor (*Fcer*^−/−^; *n* = 5), the FcγRIV (*Fcgr4*^−/−^, *n* = 5), and the FcγRIIB (*Fcgr2*^−/−^, *n* = 5), respectively, as well as wild-type C57BL/6J animals (*n* = 5) were injected with rabbit anti-Col17 IgG and EndoS-treated Col17-specific IgG (*n* = 5), respectively. EndoS, an endoglycosidase that specifically hydrolyzes the N-linked glycan on IgG heavy chains (20, 29), reduces the binding to activating FcγR, and increases the binding to the inhibiting FcγRIIB (20, 29). Disease activity was greatly reduced in *Fcer*^−/−^ and *Fcgr4*^−/−^ mice as well as in mice injected with EndoS-pretreated anti-Col17 IgG, whereas *Fcgr2*^−/−^mice showed significantly more skin lesions compared to wild-type animals as shown previously (17). MPO activity determined in extracts of an entire ear reflected disease activity and was significantly higher in *Fcgr2*^−/−^ mice (*p* < 0.001) and significantly lower in *Fcgr4*^−/−^ mice (*p* < 0.01) compared to wild-type animals (Figure 1). Of note, the intensity of bound anti-C3 and anti-C5 IgG at the DEJ as detected by direct IF microscopy of perilesional skin biopsies taken at day 12 was not different compared to wild-type animals (*Fcer*^−/−^, *p* = 0.69, *p* = 0.22; *Fcgr4*^−/−^, *p* = 0.73, *p* = 0.56; *Fcgr2*^−/−^, *p* = 0.90, *p* = 0.63; EndoS-pretreated IgG, *p* = 0.92, *p* = 0.26; Figure 1).

### Complement Activation Is Important, but Not a Prerequisite for the Development of Skin Lesions

**Figure 2:**
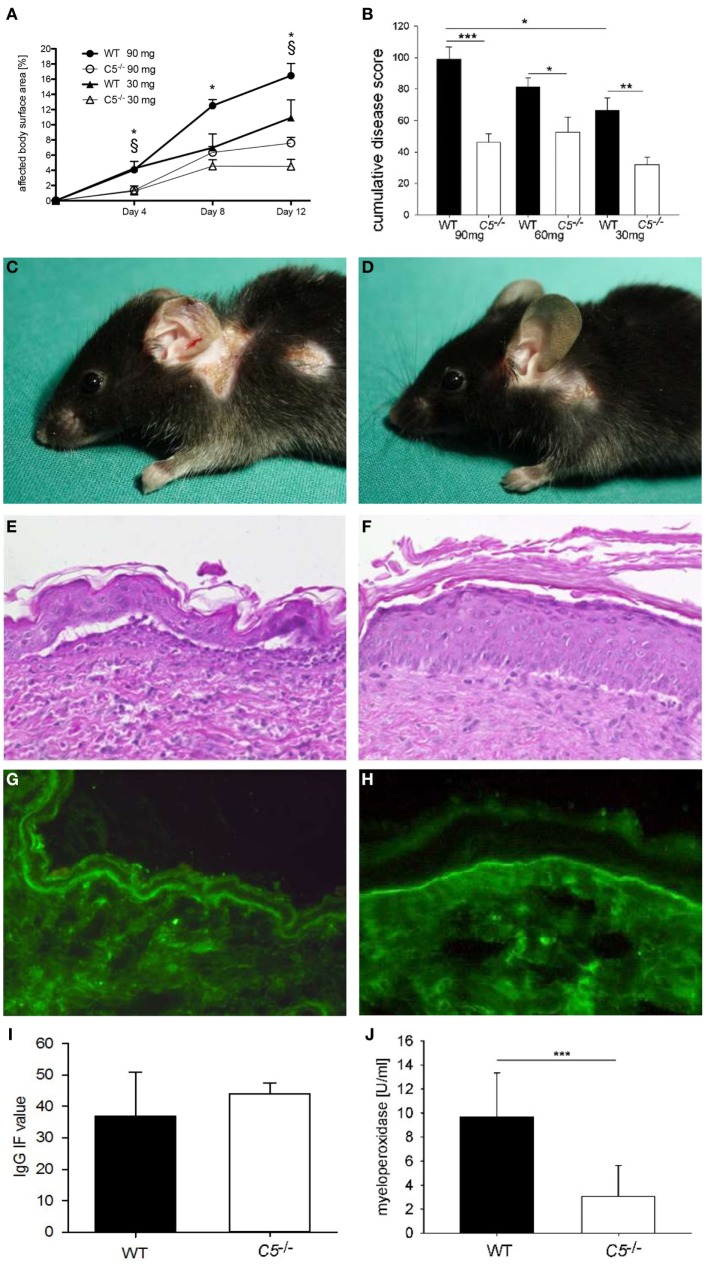
Anti-mCol17 IgG induces cutaneous disease in complement five-deficient (C5^−/−^) mice. *C5*^−/−^ (*n* = 5) and wild-type (WT; *n* = 5) mice were injected with three different doses of anti-mCol17 IgG (six times 5 mg, six times 10 mg, and six times 15 mg) every second day. Disease activity was measured as percentage of affected body surface area on days 4, 8, and 12 **(A)**. The overall clinical activity of all mice in each group was derived from the area under the curve of the affected body surface and expressed as cumulative score. The cumulative score of *C5*^−/−^ was about half compared to WT mice independent of the injected amount of anti-Col17 IgG **(B)**. Infiltration of neutrophils in lesional ear skin as reflected by myeloperoxidase activity **(J)** was significantly reduced in *C5*^−/−^ compared to WT animals. In contrast, deposits of IgG at the dermal–epidermal junction did not differ between WT and *C5*^−/−^ mice **(I)**. Representative pictures of clinical lesions **(C,D)**, lesional histopathology **(E,F)**, and IgG deposits by direct immunofluorescence microscopy **(G,H)** are shown in WT **(C,E,G)** and *C5*^−/−^ mice **(D,F,H)**. *p < 0.05, 90 mg groups; ^§^*p* < 0.05, 30 mg groups **(A)**. *p < 0.05; **p < 0.01; ***p < 0.001 **(B,J)**.

*C5*^−/−^ and corresponding wild-type mice were injected six times with 5, 10, and 15 mg of pathogenic anti-Col17 IgG, respectively (each group, *n* = 5). All mice developed cutaneous BP lesions. Disease activity in *C5*^−/−^ mice was reduced to between 36 and 54% compared to corresponding wild-type animals independent of the injected IgG concentration (30 mg IgG, *p* = 0.004; 60 mg IgG, *p* = 0.013; 90 mg IgG, *p* ≤ 0.001; Figure 2). While the disease activity was clearly dependent on the amount of injected anti-Col17 IgG (*p* = 0.025; Figures 2A,B), the extent of disease reduction in *C5*^−/−^ mice in relation to wild-type animals was independent of the anti-Col17 IgG dose (Figures 2A,B). The MPO reactivity, corresponding to the magnitude of myeloid cell infiltration, in the ears was notably higher in wild-type compared to *C5*^−/−^mice (*p* < 0.001; Figure 2J). In contrast, no difference in the linear deposits of IgG at the DEJ by direct IF microscopy of perilesional biopsies taken at day 12 was seen between wild-type and *C5*^−/−^ mice (*p* = 0.413; Figures 2G,I).

### C5aR1 Mediates the Pathogenic Effect of Anti-Col17 IgG-Induced C5 while C5aR2 Is Protective

**Figure 3:**
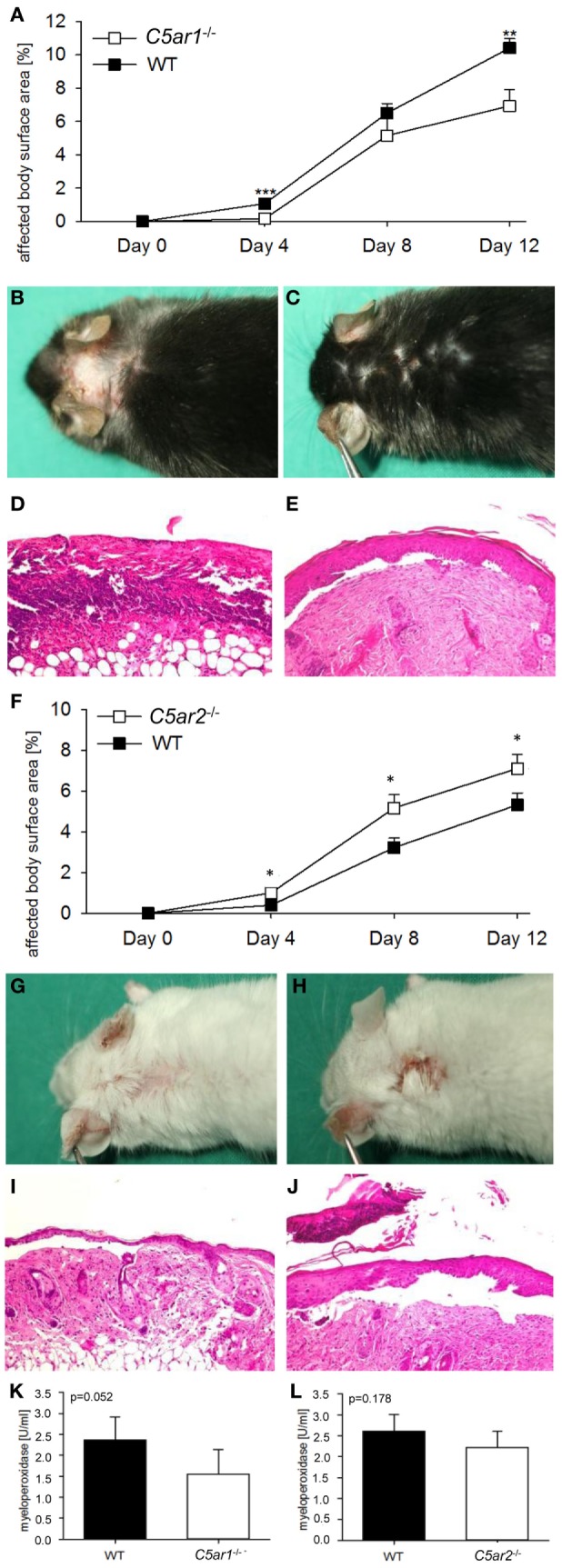
C5a receptor 1 (C5aR1) mediates tissue destruction by anti-mCol17 IgG, while C5aR2 is protective. Injection of anti-mCol17 IgG in *C5ar1*-deficient (*C5ar1*^−/−^) mice (*n* = 10) resulted in significantly less bullous pemphigoid skin lesions compared to wild-type (WT) mice [*n* = 10; **(A)**]. In contrast, injection of anti-mCol17 IgG in *C5ar2*^−/−^ mice (*n* = 15) led to a higher disease activity compared to WT mice [*n* = 18; **(F)**]. Representative pictures of clinical **(B,C,G,H)** and histopathological lesions **(D,E,I,J)** are shown in *C5ar1*^−/−^ **(C,E)**, *C5ar2*^−/−^ **(H,D)**, and WT mice **(B,D,G,I)**. Infiltration of neutrophils in skin lesions, as reflected by myeoloperoxidase activity, was reduced in *C5ar1*^−/−^ compared to WT animals [*p* = 0.052, **(K)**] and unchanged in *C5ar2*^−/−^ mice **(L)**. *p < 0.05; **p < 0.01; ***p < 0.001.

*C5ar1*^−/−^ (*n* = 10) and *C5ar2*^−/−^ mice (*n* = 15) as well as the corresponding wild-type animals (*n* = 10 and *n* = 18) were injected six times with 5 mg of pathogenic anti-Col17 IgG in two independent experiments. *C5ar1*^−/−^ mice developed less disease compared to wild-type mice. Differences were detected between the two groups when the whole observation period was considered (*p* = 0.001) as well as individually on days 4 (*p* < 0.001) and 12 (*p* = 0.007; Figures 3A–E). The difference between the two groups increased over time (*p* for interaction = 0.005). In contrast, in *C5ar2*^−/−^ mice, more skin lesions developed as compared to wild-type mice (whole observation period, *p* = 0.004) as well as on days 4 (*p* = 0.018), 8 (*p* = 0.033), and 12 (*p* = 0.046; Figures 3F–J). The difference between the two groups also increased over time (*p* for interaction = 0.017). The mean infiltration of neutrophils as determined by MPO activity in the right ears appeared to be lower in *C5ar1*^−/−^ mice (although statistical significance was not reached with *p* = 0.052) but not in *C5ar2*^−/−^animals compared to wild-type mice (*p* = 0.178; Figures 3K,L).

### Pharmacological Inhibition of C5aR Reduces Skin Lesions in a Prophylactic but Not in a Therapeutic Approach

**Figure 4:**
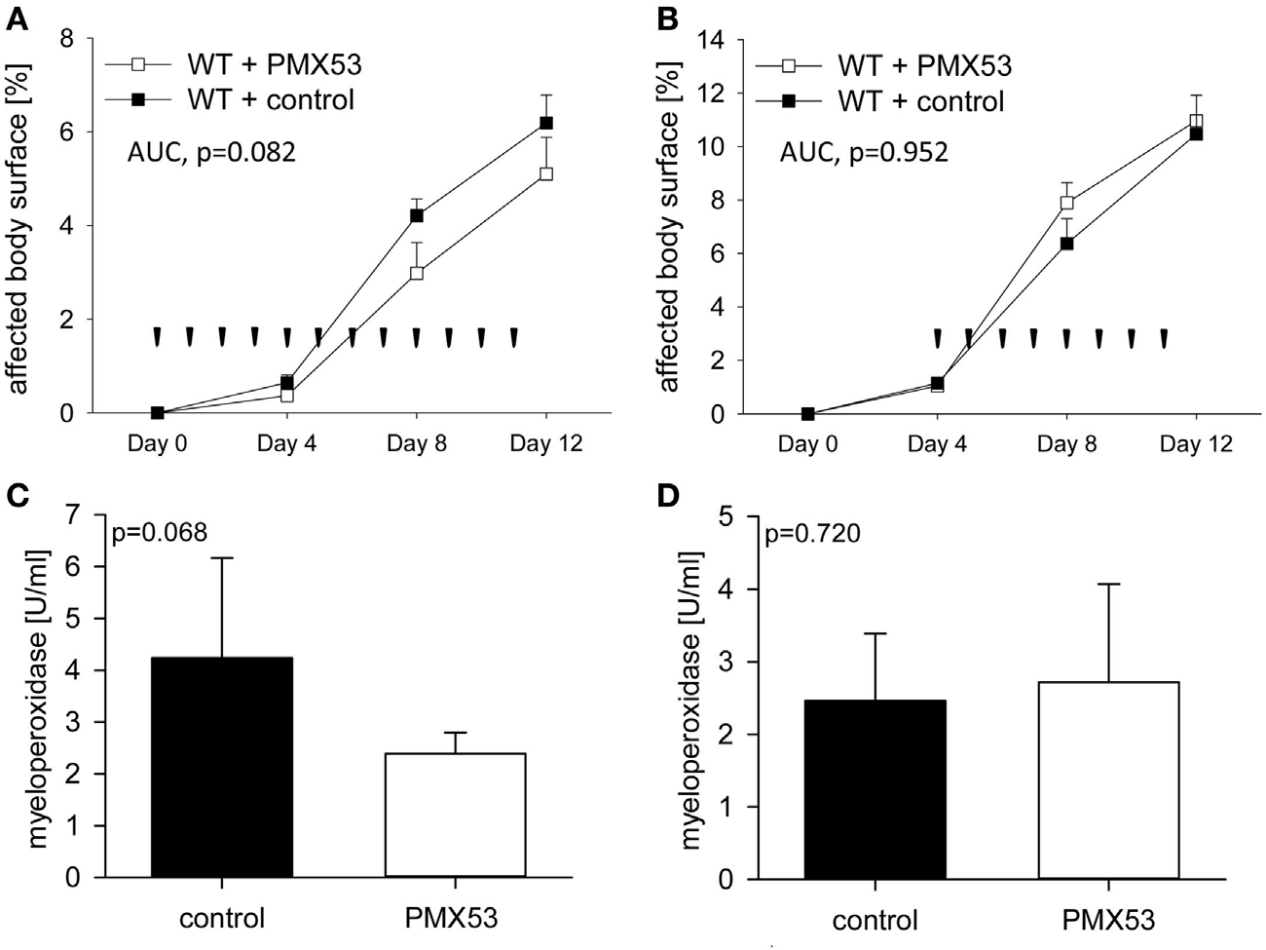
Pharmacological inhibition of C5a receptor 1 (C5aR1) reduces clinical disease in experimental bullous pemphigoid (BP) only in a prophylactic approach but not when skin lesions have already developed. When C57BL/6 mice were injected daily with the C5aR1 inhibitor PMX53 (*n* = 11) and PBS (*n* = 13), respectively, in parallel with injection of anti-mCol17 IgG on days 0, 2, 4, 6, 8, and 10, less BP lesions were observed in PMX53-treated mice **(A)**. In contrast, when PMX53 and PBS injections were started on day 4, the extent of clinical disease did not differ between the PMX53-injected and the control mice **(B)**. Arrow heads indicate injections of PMX53 and PBS, respectively. Infiltration of neutrophils in skin lesions, as reflected by myeloperoxidase activity, was reduced in the prophylactic **(C)** but not in the therapeutic approach **(D)**.

To assess the potential of C5aR1 as a new therapeutic target in BP, C57Bl/6 mice were injected six times with 5 mg of anti-Col17 IgG and received daily injections of 200 µg of the C5aR1 antagonist PMX53 (days 0–11; *n* = 11) and PBS (*n* = 13), respectively (prophylactic setting). In the PMX53-injected mice, skin lesions, disease and MPO activity in the right ears were less increased compared to control mice (although statistical significance was not reached with *p* = 0.082 and *p* = 0.068; Figures 4A,C). In a quasitherapeutic approach, daily injections of PMX53 at individual doses of 200 µg were given at day 4, when first skin lesions had already developed, and continued until day 11. In this setting, disease activity and neutrophil infiltration did not differ between PMX53-injected and control mice over time (*p* = 0.952, *p* = 0.720; Figures 4B,D).

### Complement Is Not a Main Driver of Immune Complex-Mediated ROS Release from Neutrophils

**Figure 5:**
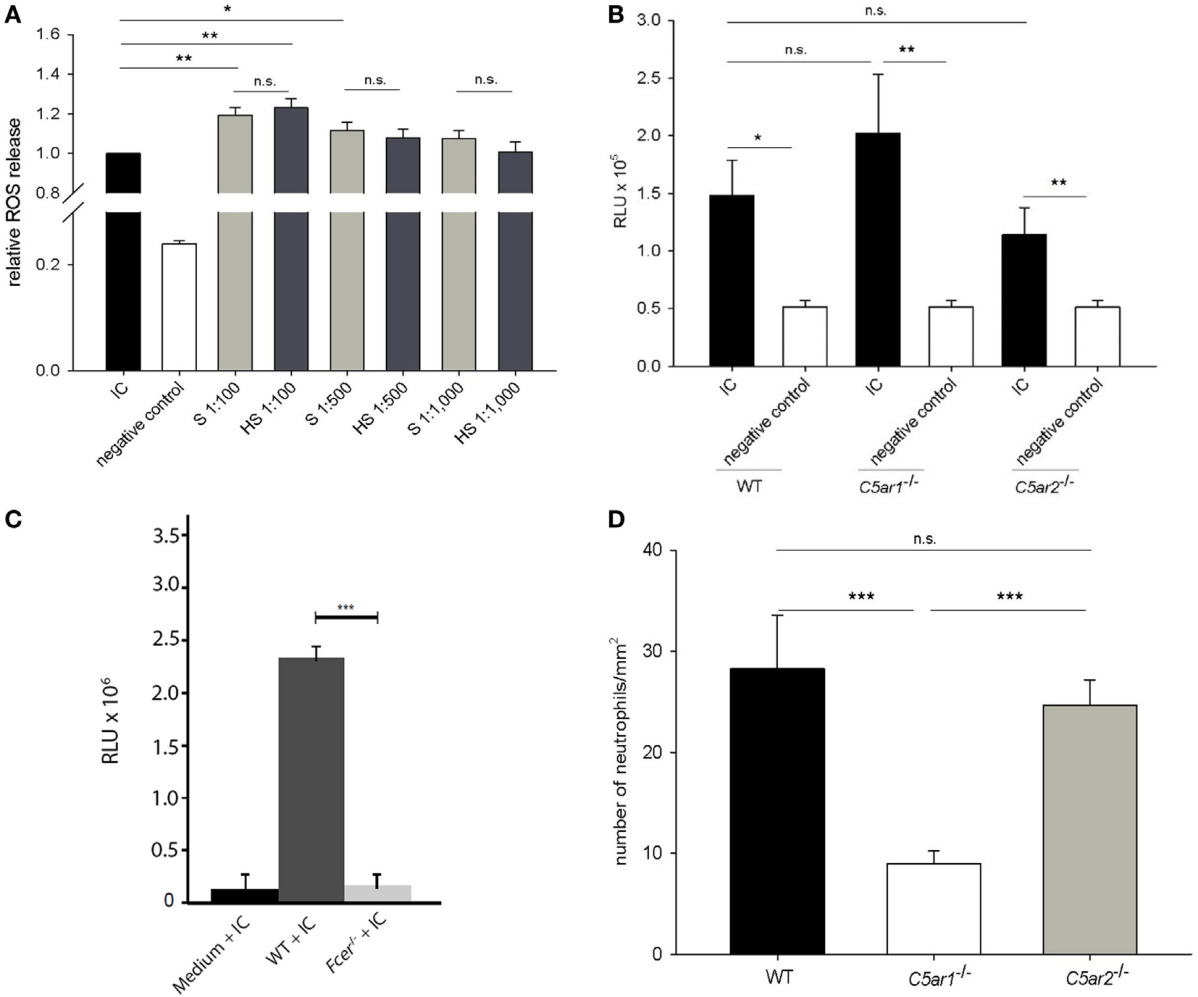
While complement activation is not the main driver of immune complex-mediated release of reactive oxygen species (ROS) from mouse neutrophils, C5a is a strong chemoattractant for neutrophils. Neutrophils isolated from the bone marrow of C57Bl/6 mice were incubated with immune complexes of mCol17 and anti-mCol17 IgG. Addition of serum (S) and heat-inactivated serum (HS), respectively, led to significantly higher ROS releases compared to stimulation with immune complexes alone, while no difference between incubation with S and HS was seen **(A)**. When neutrophils from C57BL/6 wild-type (WT), *C5ar1*^−/−^, and *C5ar2*^−/−^ mice were incubated with immune complexes, a significantly higher ROS release was seen compared to incubation with the collagen alone. No differences were observed between the ROS release of wild-type, *C5ar1*^−/−^, and *C5ar2*^−/−^ neutrophils in response to immune complexes **(B)**. In contrast, in neutrophils that lack the activating γ-chain of the Fc receptor (*Fcer*^−/−^) the ROS release was greatly reduced compared to WT cells **(C)**. Migration of bone marrow-derived neutrophils from *C5ar1*^−/−^ mice toward recombinant C5a was significantly lower compared to neutrophils from both C57BL/6 WT and *C5ar2*^−/−^ mice. No difference was seen between the migration of *C5ar2*^−/−^ and WT neutrophils. Depicted is the number of migrated cells per mm^2^ as calculated from the evaluation of ten high-power fields. PBS-treated cells served as control (not shown). Means of triplicate samples are shown **(D)**. n.s., not significant; *p < 0.05; **p < 0.01; ***p < 0.001.

The role of complement as a mediator of ROS release from neutrophils in response to immune complexes of recombinant mCol17 NC15A and anti-mCol17 IgG was explored by the use of a previously described *in vitro* assay (25, 26). In the first set of experiments, immune complexes were incubated in the presence of normal mouse serum and heat inactivated mouse serum, respectively, before mouse neutrophils were added. While the ROS release increased in response to the addition of normal mouse serum at different dilutions (*p* < 0.001), no difference in ROS release was observed between incubation with normal mouse serum and heat inactivated serum (*p* = 0.229, *p* = 548, *p* = 0.345; Figure 5A).

In the next set of experiments, we tested the involvement of C5aR1 and C5aR2 in driving the ROS release using neutrophils from wild-type, *C5ar1*^−/−^, and *C5ar2*^−/−^ mice. While clear differences were seen between immune complexes and antigen alone using wild-type, *C5ar1*^−/−^, and *C5ar2*^−/−^ neutrophils (*p* = 0.020, *p* = 0.005, and *p* = 0.008), no statistical difference was observed between the ROS release of wild-type neutrophils and neutrophils from *C5ar1*^−/−^ and *C5ar2*^−/−^ mice (*p* = 0.655, *p* = 0.554; Figure 5B). In contrast, when neutrophils from *Fcer*^−/−^ mice that lack the activating γ-chain of the Fc receptor were used, the ROS release was nearly abrogated compared to the use of wild-type neutrophils (*p* < 0.001; Figure 5C).

### The Migration of C5ar1^−/−^ but Not C5ar2^−/−^ Neutrophils Is Greatly Impaired

To test the hypothesis that neutrophil chemotaxis can be mediated by C5aRs *in vitro*, migration of wild-type neutrophils and neutrophils from *C5ar1*^−/−^ and *C5ar2*^−/−^ mice toward C5a was quantified using a modified Boyden chamber. *C5ar1*^−/−^ neutrophils showed a significantly lower migration potential compared to wild-type cells (*p* < 0.001; Figure 5D). In contrast, no difference was seen between the migration of wild-type and *C5ar2*^−/−^ neutrophils (*p* = 0.901; Figure 5D).

## Discussion

Complement can be activated by three pathways, the classical, the alternative, and the lectin pathway (30, 31). Activation of the complement system by any of the three pathways leads to activation of C3 and subsequently, to activation of C5 to form C5a and C5b. C5a is crucially involved in the host defense, immune surveillance, and tissue homeostasis (32, 33). However, C5a can also be the driver in autoimmune (34, 35) and other inflammatory diseases (36). Strong and prolonged activation of C5a leads to downregulation of immune responses in leukocytes, but has opposing effects in other cell types (37). This controversial function of C5a is often explained by the differential expression of the two C5aRs, C5aR1 and C5aR2. While signaling through C5aR1 is well characterized and reported to induce chemotaxis, mediate interaction with toll-like receptors, and regulate FcγR expression (30, 31, 38, 39), the functional properties of C5aR2 are still elusive, and both anti- and proinflammatory responses have been reported (37, 40–46).

In BP, like in various other autoantibody-mediated diseases including anti-neutrophil cytoplasmatic autoantibody-associated vasculitides, systemic lupus erythematosus, rheumatoid arthritis, antiglomerular basement membrane disease, epidermolysis bullosa acquisita, and antilaminin 332 mucous membrane pemphigoid, complement activation is regarded as pivotal for lesion formation (11–14, 19, 34, 47–51). The traditional view derived from the neonatal mouse model of BP that complement activation is required for lesion formation has recently been challenged by the observation that F(ab′)_2_ fragments of anti-hCol17 IgG also induced BP-like skin lesions in neonatal *COL17*-humanized mice (15). Complement-independent pathogenic effects of anti-Col17 antibodies have also been demonstrated *in vitro* when treatment of cultured keratinocytes with anti-Col17 antibodies led to the secretion of IL-6 and IL-8 as well as reduced cell surface expression of Col17 followed by weakened attachment of keratinocytes (52–54).

We have recently established a novel experimental model of BP in adult mice that overcame some of the shortcomings of the neonatal models, e.g., lesions develop spontaneously over some days without the application of friction (17). Importantly, in contrast to the neonatal mouse models, the novel adult mouse model was shown to be suitable to analyze the potential of anti-inflammatory agents in a quasi-therapeutic setting, i.e., in mice with already established skin lesions (17).

The two aims of the current study were therefore to clarify the pathophysiological role of complement activation by the use of the recently established BP model in adult mice and explore the therapeutic potential of complement inhibition for BP. The therapeutic potential of C5aR1 targeting is of particular relevance given the growing list of complement inhibitors that are in phase II and III clinical trials (16) and the urgent need for more specific and safe treatment options in BP. So far, long-term use of superpotent topical or oral corticosteroids is the therapeutic backbone of BP, often supplemented with potentially steroid-sparing agents such as azathioprine, methotrexate, dapsone, or doxycycline (55–58).

In previous experiments, we have shown that mice lacking the FcγR (*Fcer*^−/−^) were completely protected from the development of cutaneous disease after injection of anti-Col17, while skin lesions were significantly more extended in *Fcgr2b*^−/−^ animals (17). Identical findings were also made in an adult antibody transfer-induced model of inflammatory epidermolysis bullosa acquisita (24). When wild-type mice were injected with anti-Col17 IgG pretreated with EndoS, an endoglycosidase that specifically hydrolyzes the N-linked glycan on IgG heavy chains, only few skin lesions occurred (21, 29). Hydrolysis of IgG glycan has previously been shown to reduce the binding to activating FcγRs and thus the proinflammatory effect of autoantibodies (21). When we here quantified the intensity of C3 and C5 deposition at the DEJ at the end of the experiment on day 12, no differences between wild-type animals and mice deficient for the FcγR, FcγRIV, and FcγRIIB, respectively, and mice injected with EndoS-pretreated anti-Col17 IgG were seen. In contrast, in the same mice, significant differences in both extent of skin lesions (17) and MPO activity in ear skin, which paralleled disease activity, were seen.

A somehow similar observation was made in pemphigus, an autoimmune blistering disease characterized by autoantibodies against structural components of the desmosome, desmoglein 1 and 3 (59, 60). In pemphigus, complement deposits in the skin/epithelium is found in almost all patients, however, complement activation is not required for lesion formation (61). Our observations that complement activation at the DEJ appeared to be unrelated to the extent of clinical disease prompted us to further explore the role of complement in this novel model of experimental BP.

We then asked the question whether complement activation is a prerequisite for the induction of clinical disease in this model. When *C5*^−/−^ mice were injected with different amounts of anti-Col17 IgG, the extent of skin lesions decreased by about 50% compared to wild-type animals. Of note, the impact of C5 was independent of the anti-Col17 IgG dose. This finding is in contrast to previous findings in experimental neonatal BP in which *C5*^−/−^ mice were completely resistant to the pathogenic effect of anti-Col17 IgG (11). In fact, with *C4*^−/−^ and anti-C1q-antibody-injected mice being completely protected and factor B (*CFB*)^−/−^ mice being partly protected, both the classical and, to a lesser extent, the alternative pathway were shown to be crucial in this model (12). The importance of the classical complement pathway was also elegantly demonstrated in the *Col17*-humanized neonatal mouse model. Neonatal *Col17*^m−/−,h+^ mice injected with a monoclonal anti-human Col17 IgG1 antibody that was mutated at the C1q binding site developed less pathogenic activity compared to the unmutated antihuman Col17 IgG1-injected animals (14).

More recently, similar to our model, complement-independent pathogenic effects of anti-Col17 IgG were shown in neonatal *Col17*-humanized mice when F(ab′)_2_ fragments of anti-hCol17 IgG and anti-hCol17 IgG4, which do not activate complement at the DEJ, induced skin blistering (15, 62). In line, BP patients with predominant IgG autoantibodies and no C3 deposition at the DEJ have been described, and in 15–20% of BP patients, no C3 deposits along the DEJ can be detected (63, 64).

In subsequent experiments, we dissected the role of the two C5aRs downstream of C5 in the pathogenesis of BP and addressed the question at what stage of the disease complement activation may be important. Using knock-out mice and a specific inhibitor, we found that C5aR1 mediated the pathogenic effect of C5a, while C5aR2 appeared to protect from BP skin lesions. This is line with previous reports suggesting an anti-inflammatory role for C5aR2 counter-regulating pro-inflammatory properties of C5aR1 (42, 43). Given that the effect of C5-deficiency was more pronounced than that of C5R1-deciciency may suggest that in addition to C5aR1 and C5aR2, the membrane attack complex may exert pro-inflammatory effects. Indeed, sublytic amounts can activate the NLRP3 inflammasome (65), a mechanism that may also apply to the activation of effector cells in BP.

When we addressed the second aim of our study, i.e., the pharmacological inhibition of C5aR1, we observed reduced skin lesions after the prophylactic application of the C5aR1 inhibitor PMX53. However, this effect was weaker than that obtained with *C5ar1*^−/−^ mice. This discrepancy may be explained by an insufficient dosing or an incomplete silencing of C5aR1 by PMX. When PMX was applied in a quasi-therapeutic setting, i.e., when first skin lesions had already developed, no effect on both skin lesions and neutrophil infiltration in the dermis was seen. Combined with our results in the first set of experiments that had revealed strong complement activation along the DEJ during the course of the disease (at day 12) irrespective of the disease severity, we hypothesized that complement activation in the skin may be relevant in the early disease phase, while in established disease, complement-mediated tissue destruction may be redundant.

This hypothesis was addressed applying neutrophils, the main effector cell of tissue destruction in experimental murine BP, in two *in vitro* models. The migration assay toward C5a reflects an early disease time point when C5a is released in close vicinity of the DEJ following the attachment of anti-Col17 IgG to Col17. The ROS release assay mimics a later time point when leukocytes have already attached along the DEJ. While migration was drastically reduced in *C5ar1*^−/−^ neutrophils, no difference in the ROS release of wild-type leukocytes between stimulation with serum and heat-inactivated serum was seen. Furthermore, no significant differences in the ROS release of wild-type and *C5ar1*^−/−^ and *C5ar2*^−/−^ neutrophils were observed. In contrast, the ROS release was nearly abrogated in neutrophils from mice that lack the activating γ-chain of the Fc receptor compared to wild-type neutrophils. These results suggest that complement activation may be of particular importance in the early phase of the disease when neutrophils are attracted to the DEJ. In fact, C5a may lead to an increased expression of adhesion molecules on endothelial cells and neutrophils and can delay apoptosis (31, 66). Due to logistical constrains, cells from *C5ar2*^−/−^ mice on a C57BL/6 background were used for the *in vitro* studies. Since in previous experiments with the antibody transfer-induced mouse model of BP both wild-type and *Fcgr1*^−/−^ mice on a Balb/c background developed the same extent of clinical disease compared to animals on a C57BL/6 background (17), we believe that no principle differences between *C5ar2*^−/−^ mice on a C57BL/6 and Balb/c background in our *in vitro* studies could be expected.

Neutrophils have been identified as drivers of tissue destruction in the different mouse models of BP (6–8, 11, 18). The view that C5aR1 is important during the early phase of the disease is supported by the observation that C5aR expression on mast cells, a cell resident in the dermis, is essential for blister formation (13). Once the skin inflammation has fully developed, release of ROS and proteases from neutrophils and macrophages may become independent of complement and may be mainly mediated *via* FcγRs. This hypothesis is corroborated by previous findings in *C4*^−/−^ neonatal mice, that, although completely resistant against the pathogenic effect of anti-Col17 IgG, developed clinical blisters after injection of the neutrophil attractant IL-8 or neutrophils in the skin (12). The mechanism underlying the C5aR2-mediated anti-inflammatory effect in experimental BP requires further investigations. Two peptides with C5aR2-agonistic effect have recently been identified (37) and will facilitate this endeavor.

## Ethics Statement

Animal experiments were approved by the Animal Care and Use Committee of Schleswig-Holstein (Kiel, Germany; 21-2/11, 40-3/15) and performed by certified personnel.

## Author Contributions

TB, MH, JT, ST, FSS, and EH have performed the experiments and analyzed the data. RL, DZ, JK, CK, and ES have designed the work and interpreted the data. FS and ST generated the figures. IK has interpreted the data and performed the statistical analyses. CK and ES have, in addition, overseen the experimental work and written the manuscript. All authors have approved the final version of the manuscript.

## Conflict of Interest Statement

The authors declare that the research was conducted in the absence of any commercial or financial relationships that could be construed as a potential conflict of interest.
